# A Continuously Tunable Full-Color Emission Nitrogen-Doped Carbon Dots and for Ultrasensitive and Highly Selective Detection of Ascorbic Acid

**DOI:** 10.3390/nano12040693

**Published:** 2022-02-19

**Authors:** Demin Huang, Haiyan Qi, Jing Jing, Rokayya Sami, Tao Jing, Sultan J. Alsufyani, Nada Benajiba, Nawal Madkhali

**Affiliations:** 1 College of Chemistry and Chemical Engineering, Qiqihar University, No. 42, Wenhua Street, Qiqihar 161006, China; huang15846285980@sina.com (D.H.); jtkr@163.com (T.J.); 2 School of Medicine and Health, Harbin Institute of Technology, No. 92, West Dazhi Street, Harbin 150000, China; 3 Department of Food Science and Nutrition, College of Sciences, Taif University, P.O. Box 11099, Taif 21944, Saudi Arabia; 4 Department of Physics, College of Sciences, Taif University, P.O. Box 11099, Taif 21944, Saudi Arabia; s.alsufyani@tu.edu.sa; 5 Department of Basic Health Sciences, Deanship of Preparatory Year, Princess Nourah bint Abdulrahman University, P.O. Box 84428, Riyadh 11671, Saudi Arabia; nabenajiba@pnu.edu.sa; 6 Department of Physics, College of Sciences, Imam Mohammad Ibn Saud Islamic University (IMISU), Riyadh 11623, Saudi Arabia; namadkhali@imamu.edu.sa

**Keywords:** nitrogen-doped carbon dots, full-color emission, ascorbic acid, fluorescence detection

## Abstract

Nitrogen-doped carbon dots exhibiting excitation-dependent full-color emissions (F-NCDs) were prepared via the one-step hydrothermal method with citric acid and phenylenediamine. Specifically, the emission wavelength of the F-NCDs tuned from 452 nm to 602 nm due to the introduction of new energy levels by C=O and C=N functional groups. We exploited its stability in illumination, ionic strength, and pH, as well as its specificity, sensitivity, especially in ascorbic acid (AA) detection. F-NCDs could measure the AA concentration in the linear ranges of 0~0.1 and 0.1~1 mmol/L with the detection limit (LOD, S/N = 3) as low as 2.6 nmol/L. Additionally, we successfully detected AA in bovine serum with our F-NCDs and obtained the result within 1 min. Because of full-color emission features, we believe our F-NCDs have a great potential in fluorescent sensor detection.

## 1. Introduction

Carbon dots (CDs), which are fluorescent nanoparticles with particle size less than 10 nm, have excellent optical properties and high stability. Moreover, they have several advantages, including simple synthesis, low toxicity, easy modification, good biocompatibility, and good water solubility. Therefore, they have been widely used in many fields, such as drug-loaded therapy [1], bioimaging [2,3,4,5,6], sensors [7], medical diagnosis [8,9] and printing inks [10]. Previous studies focused on their excitation-dependent vs. the excitation-independent features. However, most CDs show their emissions in the blue to green-light regions, which significantly restrict their biological applications. In recent years, the full-color emissions CDs (F-CDs) have been discovered, which display continuously tunable excitation-dependent full-color emission with reliable intensity. With an unaltered chemical structure, they can widely integrate with photoluminescence body and build versatile sensing systems by ignoring the matching of energy gaps. Therefore, it is of great significance to attain the F-CDs for further research and application, particularly in the biological field.

Ascorbic acid (AA) is not only one of the essential nutrients for the human body, but also a good natural antioxidant which has been used as an additive in many commercial products. As one of the essential nutrients, AA plays vital roles in metabolism, body growth, development [11,12,13], antibody formation, iron absorption [14], folic acid and vitamin E stabilization. By scavenging free radicals, AA can prevent cardiovascular diseases, diabetes, and cancer. Furthermore, AA deficiency even causes cold, scurvy, atherosclerosis, and oxidative damage to lipids, proteins, and DNA [15]. However, excess AA also leads to adverse reactions, including thrombosis, diarrhea, distention, urinary tract, and kidney infection. Therefore, there is an urgent demand to monitor AA concentration. Currently, various methods, including redox titration [16], high performance liquid chromatography (HPLC) [17], electrochemical [18] and fluorescence spectrophotometry analysis [19,20] have been used to detect AA concentration. Considering their excellent optical and chemical properties, CDs could also serve as an ideal fluorescent material to monitor AA concentration with low cost, easy and rapid detection, and high sensitivity and accuracy.

Herein, we present a nitrogen-doped full-color emissions carbon dots (F-NCDs) for ascorbic acid (AA) detection using citric acid and *p*-phenylenediamine as raw material. According to detailed structure and properties characterizations, we postulated that the C=O and C=N functional groups imported new energy levels for electronic conversion and generated the continuously tunable excitation-dependent full-color emissions. Furthermore, we successfully applied F-NCDs as a fluorescence sensor to detect ascorbic acid (AA) in serum.

## 2. Materials and Methods

### 2.1. Chemicals and Reagents

Citric acid was purchased from Tiantian Chemical Reagent Factory (Tianjin, China). *p*-phenylenediamine was obtained from Aladdin Reagent Co., Ltd. (Shanghai, China). Phenylalanine, arginine, glycine, threonine, aspartate, tryptophan, glutamate, and tyrosine were purchased from Tianjin Institute of Fine Chemical Industry Co., Ltd. (Tianjin, China). KCl, MgCl_2_, CaCl_2_, NaCl, MnSO_4_, SrCl_2_, CdSO_4_, NiSO_4_, and Fe_2_(SO_4_)_3_ were obtained from Tianjin Kaitong Chemical Reagent Co., Ltd. (Tianjin, China). CuSO_4_, HgCl_2_, PbSO_4_, ZnCl_2_, AlCl_3_, HCl, NaOH, and anhydrous ethanol were acquired from Beijing Chemical Plant (Beijing, China). Quinine sulfate was acquired from Beijing Enoch Technology Co., Ltd. (Beijing, China). All chemicals were analytical pure, and the experimental water was ultrapure water. Fetal bovine serum was obtained from Zhejiang Tianhang Biotechnology Co., Ltd. (Zhejiang, China).

### 2.2. Synthesis of F-NCDs

**Figure 1:**
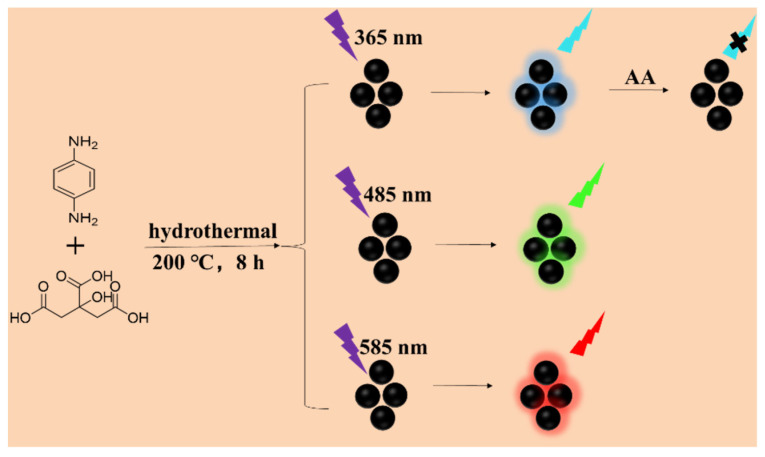
F-NCDs synthesis pathway.

F-NCDs were synthetized using a hydrothermal synthesis method. As shown in Figure 1, citric acid and *p*-phenylenediamine were dissolved in 10 mL ultrapure water with a mass ratio of 2.5:1. Then, the mixture was autoclaved and left to react at 200 °C for 8 h. After reaction, the sample was cooled naturally to room temperature. Later, the yellow-brown solution to be dialyzed was placed in a sealed dialysis membrane (MWCO = 500 Da) and immersed in ultrapure water for 64 h. After dialysis, solutions were frozen and light yellow carbon dot powder was obtained, named F-NCDs. Furthermore, 5 mg F-NCDs powder was dissolved in 5 mL ultrapure water to create a 1 mg/mL F-NCDs solution.

### 2.3. Characterization of F-NCDs

A JEM-2100F transmission electron microscope (Tokyo, Japan) was used to acquire high-resolution transmission electron microscope (HRTEM) images of F-NCDs.

The D8-FOCUS X-ray powder diffractometer (Waltham, MA, USA) was applied to measure the XRD spectrum of F-NCDs.

Additionally, the ESCALAB 250Xi X-ray photoelectron spectrometer (Waltham, MA, USA) was used to analyze the composition element and surface functional groups of F-NCDs.

The NICOLET 380 spectroscopy (Waltham, MA, USA) was employed to acquire the Fourier transform infrared (FTIR) spectrum of F-NCDs with KBr particles as the background, with a spectral resolution of 4 cm^−1^ and scanning range between 4000~500 cm^−1^.

The TU-1901 UV–vis spectrophotometer (Beijing, China) was utilized to measure the UV–vis spectra of F-NCDs. F-NCDs Spectra were acquired with xenon lamps which automatically converts excitation wavelengths as a light source for testing, scan range between 200~600 nm, 2 mm spectral bandwidth, 2.0 nm variable slit, 1 nm scan interval, at medium scanning speed.

Shimadzu RF4301-PC Fluorescence Spectrometer (Kyoto, Japan) was employed to measure the fluorescence spectra of F-NCDs. All optical tests were conducted at room temperature. The conditions were set as follows: the excitation wavelength at 325 nm (λ_max_ = 325 nm), scanning range between 300~600 nm, incident slit width at 5 nm, shot slit width at 5 nm, medium scan speed.

Finally, a FLUOROMAX-4 high sensitivity fluorescence spectrometer (Kyoto, Japan) was used for researching the fluorescence lifetime of F-NCDs.

### 2.4. Detection of AA

To detect ascorbic acid, 100 μL F-NCDs solution and 2 mL Tris HCl buffer (pH = 7.4) were mixed. Various concentrations of AA solution were added and then the spectra were recorded after 60 s.

## 3. Results and Discussion

### 3.1. Characterization of F-NCDs

**Figure 2:**
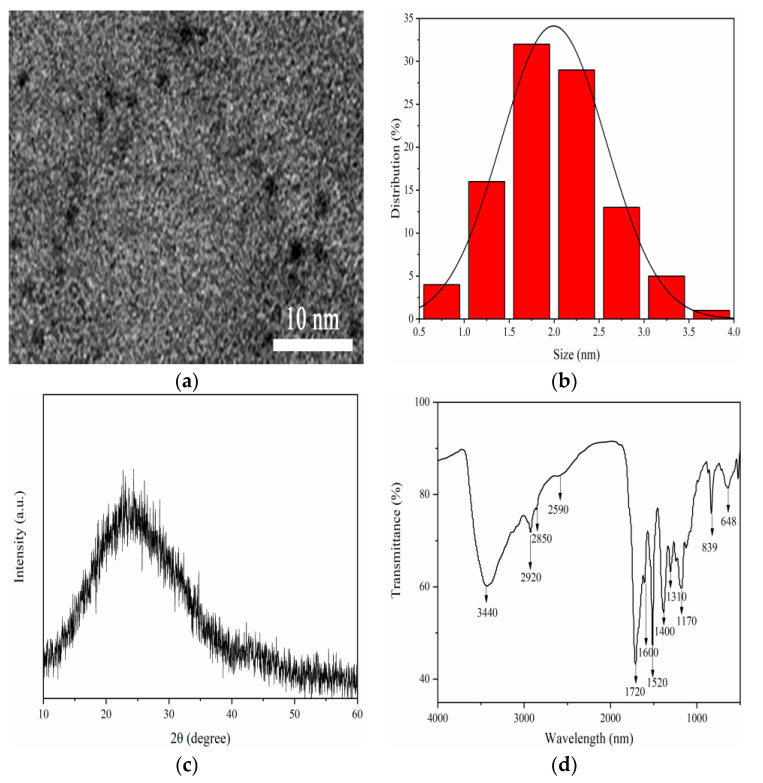
(**a**) HRTEM image of F-NCDs; (**b**) Size distribution of F-NCDs; (**c**) XRD spectrum of F-NCDs; (**d**) FTIR spectrum of F-NCDs.

The HRTEM image of F-NCDs showed that the particles were approximately spherical (Figure 2a), with size distribution between 0.7~3.5 nm, and the average size of 2.0 nm (Figure 2b). The XRD spectrum of F-NCDs showed that there was an obvious diffraction peak around 23° (Figure 2c), which corresponded to the amorphous structure [21,22]. FTIR spectrum of F-NCDs displayed that a typical wide absorption band emerged at 3000~3500 cm^−1^ which corresponded to N–H, O–H and unsaturated C–H stretching vibrations [23,24,25]. The peaks at 2929 cm^−1^ and 2850 cm^−1^ were caused by saturated C–H bond stretching vibrations. The absorption bands at 1660~1720 cm^−1^ were related to C=O and C=N stretching vibrations. The shoulders between 1600~1400 cm^−1^ were related to C=C stretching vibration, indicating the possible presence of a benzene ring structure in F-NCDs. In addition, three obvious bands at 1310 cm^−1^, 1170 cm^−1^ and 839 cm^−1^ were correlated to C–O stretching vibration, C–N stretching vibration, and N−H deformation vibration, respectively (Figure 2).

**Figure 3:**
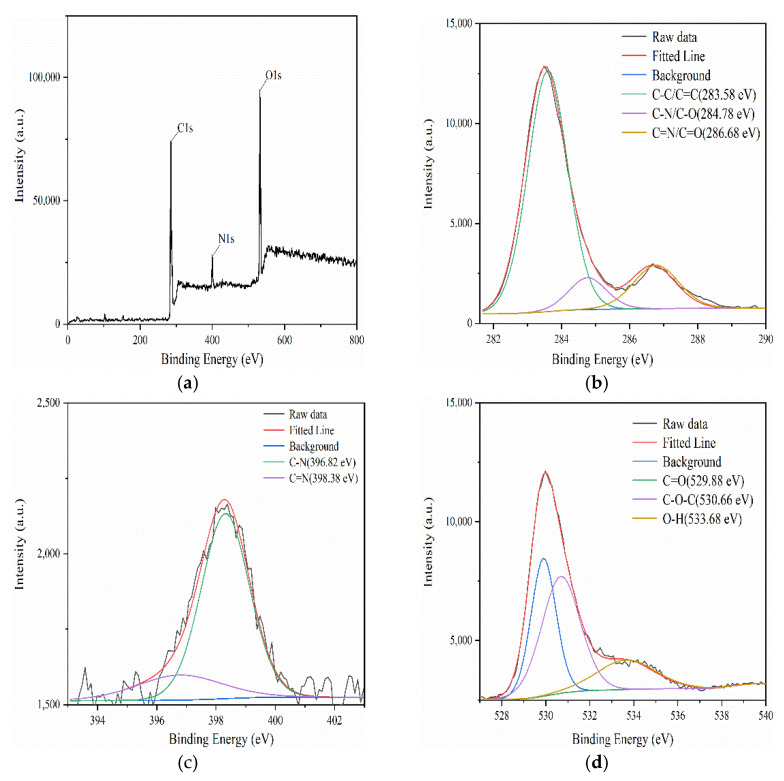
(**a**) XPS spectrum of F-NCDs; (**b**) C1s high-resolution XPS pattern of F-NCDs; (**c**) N1s high-resolution XPS pattern of F-NCDs; (**d**) O1s high-resolution XPS pattern of F-NCDs.

To reveal more detailed information about the functional groups of F-NCDs, we performed an XPS analysis. Full XPS survey spectrum showed the characteristic peaks of C1s, N1s and O1s at 285.19, 398.38 and 532.17 eV, respectively (Figure 3a). A further elemental analysis indicated the composition ratio of C, N and O were 65.95%, 7.7% and 24.55%, respectively. The C1 characteristic peaks were decomposed into three peaks at 283.58, 284.78 and 286.68 eV, which corresponded to the functional groups of C–C/C=C, C–N/C–O and C=N/C=O, respectively (Figure 3b). The decomposition peaks of N1s were at 396.82 and 398.38 eV, which correlated with C–N and C=N bond, respectively (Figure 3c). O1s characteristic peaks displays three peaks at 529.88 eV, 530.66 eV and 533.68 eV, which matched with C=O, C–O–C, and O–H, respectively [26,27,28,29] (Figure 3d). In conclusion, we speculated that the surface of F-NCDs might contain –OH, –COOH, –NH_2_, benzene ring, imine and carbonyl groups, indicating that it can be easily modified and has a good water solubility.

### 3.2. Optical Characteristics of F-NCDs

**Figure 4:**
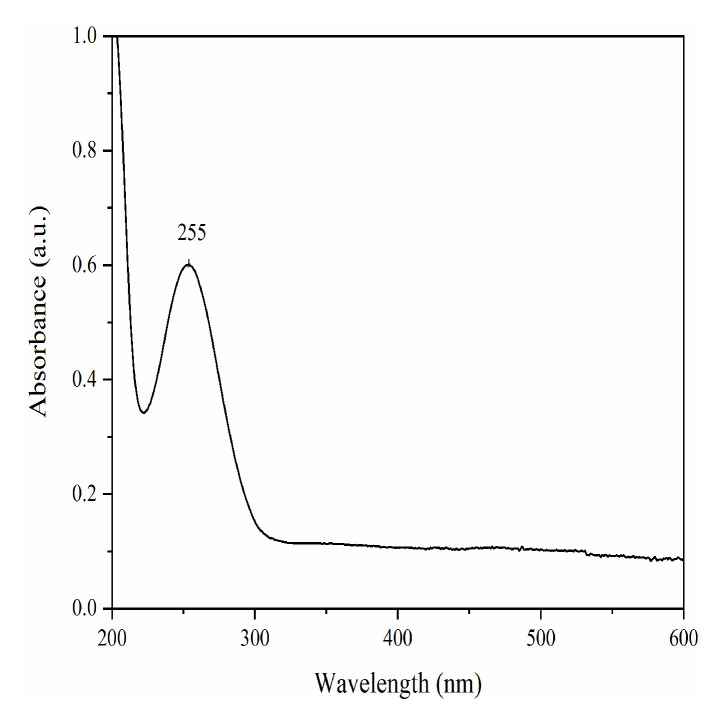
UV spectra of F-NCDs.

The UV-absorption spectrum of F-NCDs showed a strong absorption peak at 255 nm, suggesting the existence of a B absorption band (π→π*), correlated to the presence of a benzene ring (Figure 4). Furthermore, a constant intensity of absorption was observed in the range of 300~600 nm. Fluorescent quantum yield, measured with comparative method detection [30], was 67.59% at 345 nm excitation, which using quinine sulfate (QY = 54%) in 0.1 mol/L H_2_SO_4_ as the reference.

**Figure 5:**
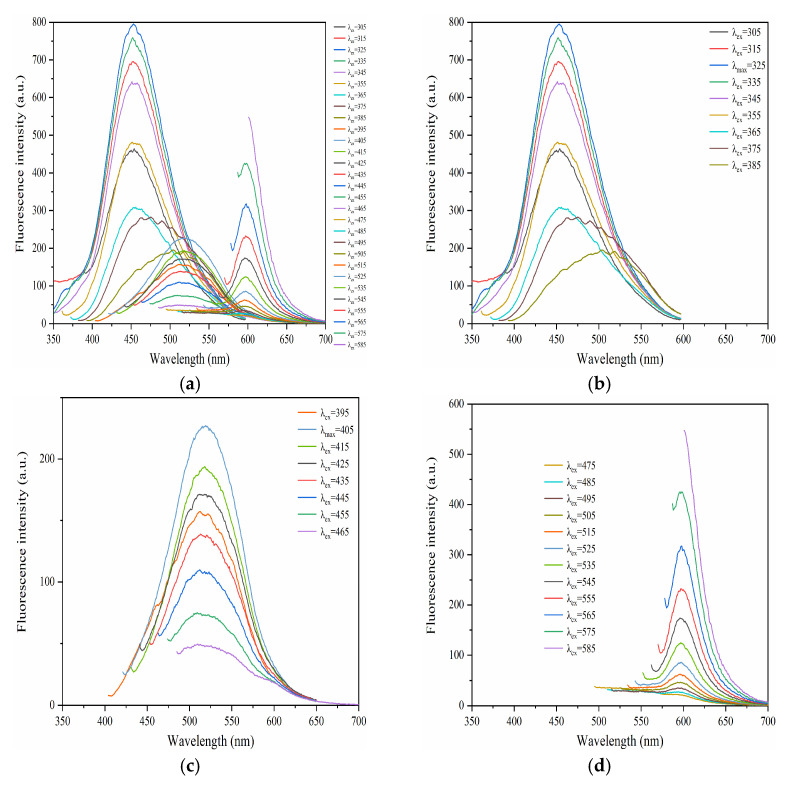
(**a**) Fluorescence spectra of F-NCDs under various excitation wavelengths (305~585 nm); (**b**) Fluorescence spectra under the excitation wavelengths between 305~385 nm; (**c**) Fluorescence spectra under the excitation wavelengths between 395~465 nm; (**d**) Fluorescence spectra under the excitation wavelengths between 475~585 nm.

We decided to examine the excitation and emission properties of F-NCDs afterwards. The F-NCDs exhibited nearly continuous excitation-dependent emission. The emission wavelengths of F-NCDs were dependent on the excitation wavelengths ranging from 305 nm to 585 nm (Figure 5a). Interestingly, the emission spectra were divided into three parts and we noticed three distinct emission peaks of F-NCDs at 452 nm, 517 nm and 602 nm (Figure 5a). When we examined them individually, we found these emission peaks corresponded to an excitation wavelength between 305~385 nm, 395~465 nm and 475~585 nm, respectively (Figure 5b–d).

**Figure 6:**
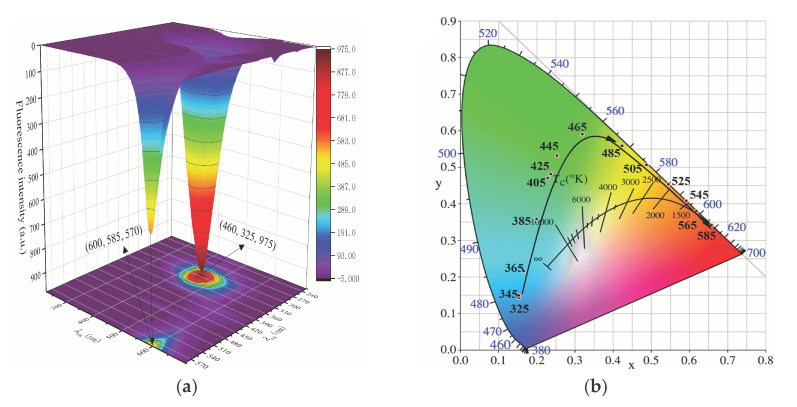
(**a**) Excitation-emission 3D matrix of F-NCDs; (**b**) CIE chromaticity coordinates of F-NCDs.

To further investigate the tunable change of color under the excitation-dependent fluorescence, we performed 3D fluorescent matrix scans on F-NCDs. It had a broad emission spectrum ranging from 220 to 700 nm, with the excitation wavelengths ranging from 325 to 585 nm with 20 nm increments (Figure 6a). As expected, with a red-shift of the excitation wavelength, the emission spectrum displayed three fluorescence centers. The emission centers at 452 nm and 602 nm were obvious; however, the center at 517 nm was unnoticeable, possibly due to the low fluorescence intensity.

CIE color coordinates from fluorescent matrix scans of F-NCDs gradually moved from blue to cyan, green, yellow, orange, and eventually red region under different excitation wavelengths displaying from 325 to 585 nm (Figure 6b). Detailed photoluminescence properties of F-NCDs, including λ_em_, λ_ex_, Δλ (λ_em_ − λ_ex_), fluorescence intensity, and CIE color coordinates are listed in Table 1. From the results, we observed the irregular change of the fluorescence intensity and Δλ (Table 1), suggesting the photoluminescence properties of F-NCDs contributed to multivariate surface states.

### 3.3. Full-Color Emission Mechanism of F-NCDs

**Figure 7:**
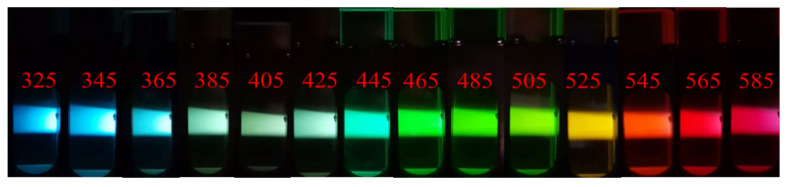
Fluorescent images of F-NCDs at different excitation wavelengths.

Optical images of F-NCDs were obtained with excitation-dependent full-color emission and ranged from blue to red (Figure 7). Remarkably, F-NCDs demonstrated selective emission in a wide color range. From these images, we directly observed the fluorescent color changed from blue to cyan, cyan to green, green to yellow, yellow to reddish orange and finally turns red, when the excitation wavelengths ranged from 325~385 nm, 385~465 nm, 465~505 nm, 505~545 nm and 545~585 nm. This information was consistent with the fluorescent color change in the CIE color result.

**Figure 8:**
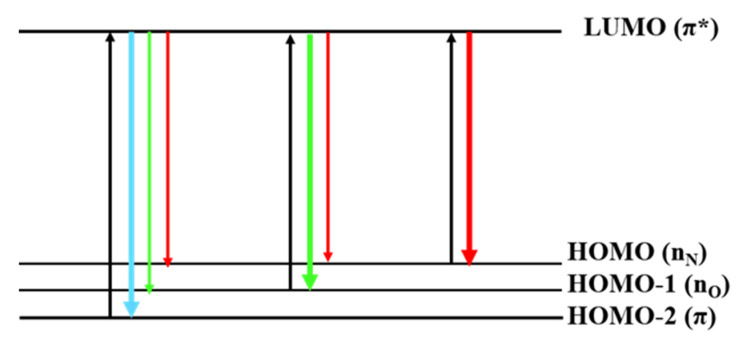
Proposed full-color emission mechanism of F-NCDs.

Subsequently, we decided to study potential factors affecting the full-color emission characteristics of F-NCDs. Previous studies suggested that the quantum-size effect, surface states and molecular states might have influenced the emission characteristics of carbon dots [31,32]. We speculated that the full-color emission properties of F-NCDs were determined by its structure, for instance the functional groups C=O and C=N on its surface. Both functional groups produced rich structural arrangements and imported new energy levels into their electronic structures, causing more electronic conversion probabilities [33]. We illustrated the possible energy levels of F-NCDs (Figure 8). For the F-NCDs, HOMO-1 and HOMO energy levels emerged as a result of the introduction of C=O, C=N groups. Then, electron transitions could occur from the two new HOMO-1 and HOMO to the LUMO (π*); meanwhile, the excited electrons returned to HOMO-1, or HOMO levels by radiative transition, leading to fluorescence in the green and red regions. When the electrons absorbed short-wave light, electron transitions occurred from HOMO-2, with an energy level to the LUMO (π*) level. Then, the excited electrons returned to HOMO-2, HOMO-1, or HOMO levels by radiative transition, and, therefore, caused the broad fluorescence emission of F-NCDs from blue to red region.

### 3.4. Application of F-NCDs

To examine the potential application of F-NCDs, we measured its fluorescence stability and analytical performance. To achieve the best specificity and sensitivity, we examined the fluorescence emission spectrum of F-NCDs at 325 nm excitation.

#### 3.4.1. Stability of F-NCDs

**Figure 9:**
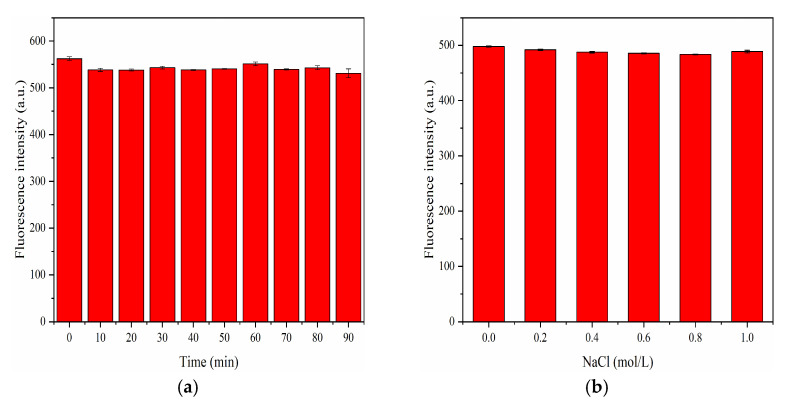
(**a**) Fluorescence stability of F-NCDs under 365 nm UV light; (**b**) Fluorescence stability of F-NCDs under various ionic strengths.

To investigate the fluorescence stability of F-NCDs, we explored the fluorescent emission peak under various conditions, including illumination, ionic strength, and pH. After 90 min of continuous UV light irradiation, the fluorescence intensity of F-NCDs remained stable, showing good fluorescence stability (Figure 9a). When we examined the impact of ionic strength (NaCl), the fluorescence intensity of F-NCDs did not show significant change, with an ionic strength up to 1.0 mol·L^−1^, indicating its tolerance to ionic change (Figure 9b). Above all, we confirmed that F-NCDs had stable and excellent optical performance even under extreme environmental conditions, indicting its significant potential for sensor application in physiological environment.

**Figure 10:**
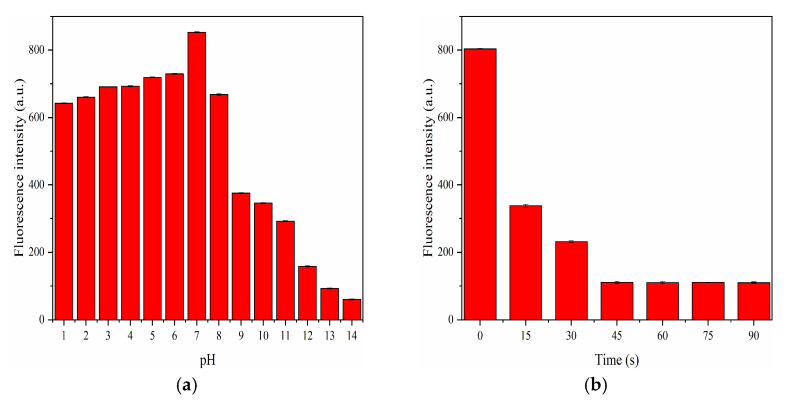
(**a**) Fluorescence intensity of F-NCDs under different pH; (**b**) Response time of F-NCDs with the presence of AA.

Since pH is a key factor in actual detection, it would be very important to examine the fluorescence stability of F-NCDs under different pH conditions. The fluorescence intensity gradually increased with an increasing pH value from 1 to 6, then greatly increased with an increasing pH from 6 to 7, and finally reached the maximum at pH 7 (Figure 10a). However, the intensity dramatically decreased when the pH value rose from 8 to 14, and it was almost completely quenched with pH value 13 and 14. In conclusion, we postulated that F-NCDs had a good fluorescence stability in an acidic and neutral environment, suggesting its utilization in vivo.

Ascorbic acid (AA) interacted with F-NCDs and could quench its fluorescence, we decided to explore its response time when AA was present. As expected, when AA was added, the fluorescence intensity decreased remarkably within 15 s and continued to decline within 45 s; however, after 45 s, the fluorescence intensity was kept constant until 90 s (Figure 10b). To ensure the stability of F-NCDs, 1 min was used as the appropriate reaction time for the following experiments when detecting AA.

#### 3.4.2. Detection of Ascorbic Acid (AA)

**Figure 11:**
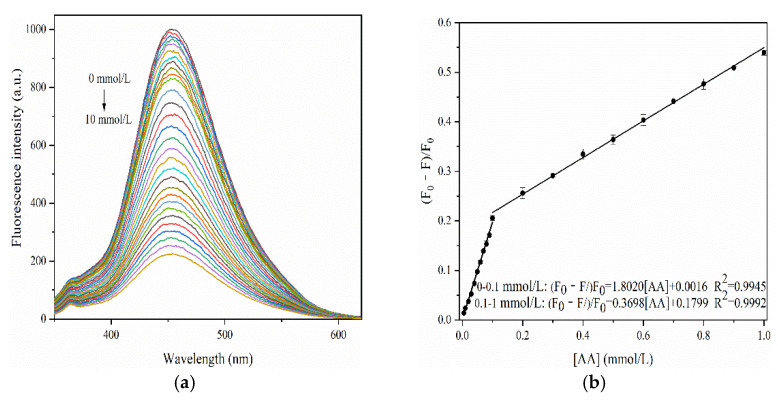
(**a**) Fluorescence emission spectra of F-NCDs in the presence of AA; (**b**) the relationship between fluorescence change ((F_0_ − F)/F_0_) and AA concentrations.

To evaluate the response of F-NCDs to AA, we examined its fluorescent emission intensity under pH 7. With rising AA concentrations from 0 mM to 10 mM, the fluorescence strength of F-NCDs gradually decreased (Figure 11a). The relationship between (F_0_ − F)/F_0_ and AA concentration was calculated, where F and F_0_ represent the fluorescence strength of F-NCDs with and without AA, respectively (Figure 11b). In the range of 0~0.1 mM and 0.1~1 mM, it showed a perfect linear relationship, fitting linear equations (F_0_ − F)/F_0_ = 1.8020[AA] + 0.0016 (R^2^ = 0.9945) and (F_0_ − F)/F_0_ = 0.3698[AA] + 0.1799 (R^2^ = 0.9992), respectively. The lowest detection limit (LOD, S/N = 3) of AA was 26 nM, indicating its sensitivity for AA detection. Moreover, we compared our F-NCDs results with previous studies and concluded that only our F-NCDs could detect extremely low AA concentration (Table 2).

**Figure 12:**
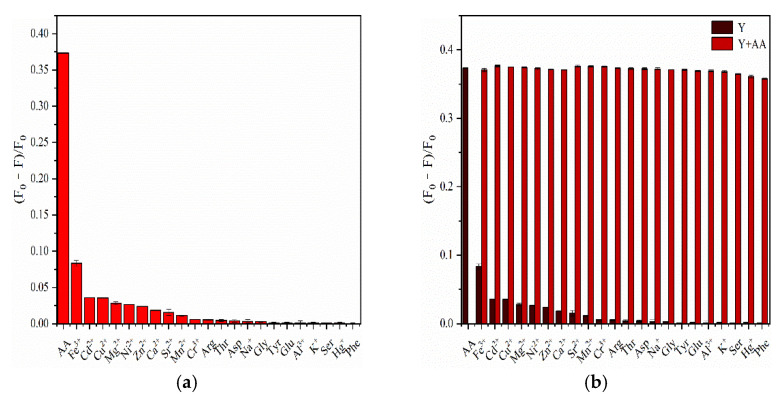
(**a**) Effects of different metal ions and amino acids on fluorescence intensity change of F-NCDs. AA served as a control; (**b**) Effects of different metal ions and amino acids on fluorescence intensity change of F-NCDs with and without AA.

To evaluate the specificity of F-NCDs to AA detection, we investigated their performances to various metal ions (Fe^3+^, Cd^2+^, Na^+^, Al^3+^, Cu^2+^, Cr^3+^, Mg^2+^, Zn^2+^, Ni^2+^, Ca^2+^, Sr^2+^, Mn^2+^, K^+^, Hg^2+^) and amino acids (arginine (Arg), threonine (Thr), aspartic acid (Asp), glycine (Gly), tyrosine (Tyr), glutamic acid (Glu), serine (Ser) and phenylalanine (Phe)). Compared to other ions and amino acids, only AA exhibited the greatest change in the fluorescence intensity of F-NCDs, indicating the high selectivity of F-NCDs for AA detection (Figure 12a). Furthermore, when interference substances coexisted with AA, AA still dominated the change in F-NCDs, and the effect caused by the coexistent interference substance could be neglected, further suggesting the specificity of F-NCDs for AA detection (Figure 12b).

#### 3.4.3. Mechanism for Ascorbic Acid (AA) Detection

**Figure 13:**
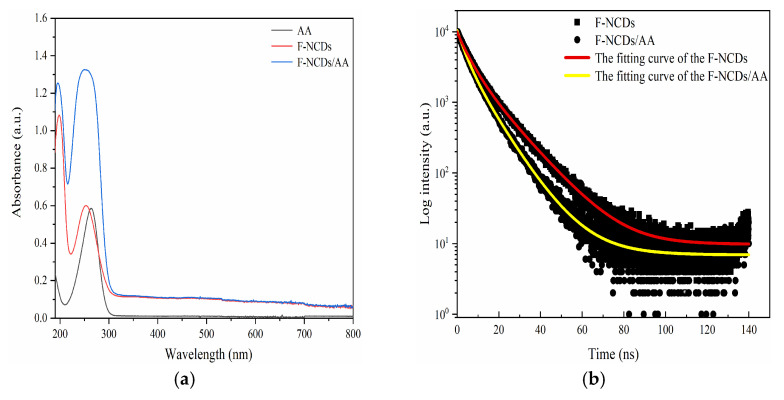
(**a**) UV-vis spectrum of AA only, F-NCDs only, and F-NCDs with AA; (**b**) Fluorescence decay curves of F-NCDs with or without AA.

To explore how AA quenched the fluorescence of F-NCDs, we performed UV absorption and fluorescence lifetime experiments. The absorption peak intensity of F-NCDs at 255 nm increased when AA was added. However, no additional absorption peak was observed (Figure 13a). When we measured the average fluorescence lifetime of F-NCDs, the calculated average (amplitude-weighted) lifetime was reduced from 10.2 ns to 7.68 ns with the addition of AA, suggesting that the presence of AA reduced the average lifetime of F-NCDs significantly (Figure 13b). Above all, these findings revealed that the fluorescence quenching mechanism of F-NCDs/AA system was dynamic [30,41,42].

#### 3.4.4. Detection of Ascorbic Acid (AA) in Bovine Serum

To further investigate the feasibility and practical application of F-NCDs, we decided to detect ascorbic acid in the bovine serum. We calculated the original AA concentration as 42.28 μmol/L (RSD = 0.20) in animal serum, which was consistent with a previous report. Additionally, three different concentrations (20, 30, 40 μmol/L) of AA were added into the serum and the performance of our F-NCDs was evaluated. As expected, the recovery rate was as high as 99.89~101.54% (RSD < 2.1%) (Table 3). In conclusion, we proved the practicability and accuracy of our F-NCDs in detecting the concentration of AA in animal serum.

## 4. Conclusions

In this study, we developed nitrogen-doped full-color emissions carbon dots through a one-step hydrothermal method. The surface states of F-NCDs contributed its tunable excitation-dependent full-color emissions. Our F-NCDs exhibited excellent optical properties, fluorescence intensity and stability. Additionally, based on the dynamic quenching mechanism, the F-NCDs could detect AA in serum with decent results. Compared with other sensors, our F-NCDs do not require any surface modification, and thus it could perform simple, efficient, and direct detection. Overall, our work achieved significant advances in the excitation-dependent full-color emissions of carbon dots, and we believe our F-NCDs could be applied in many areas for detection.

## Figures and Tables

**Table 1:** Photoluminescence properties of F-NCDs.

λ_ex_/nm	λ_em_/nm	△λ/nm	Fluorescence Intensity	x	y
325	452	127	795	0.1533	0.1425
345	455	110	642	0.1533	0.1487
365	466	101	431	0.1662	0.2165
385	505	120	216	0.1995	0.3590
405	517	112	227	0.2287	0.4705
425	520	95	171	0.2370	0.4819
445	523	78	109	0.2537	0.5324
465	526	61	49	0.3203	0.5905
485	593	108	31	0.4242	0.5591
505	598	93	46	0.4880	0.5069
525	599	74	85	0.5449	0.4537
545	600	55	174	0.5920	0.4074
565	601	36	317	0.6192	0.3803
585	602	17	600	0.6584	0.3413

**Table 2:** Performance comparison of different methods for AA detection.

Materials Used	Method	Method APPLIED	linear Range (μmol/L)	LODs (μmol/L)	Ref.
acriflavine	spectrofluorometric method	fluorometric	11,400~56,800	454	[34]
PAP/ZrO_2_NPs/CNTs/GCE	voltammetric	electrode	1~295	0.35	[35]
boron doped diamond	voltammetric	electrode	18.5~370	5.4	[36]
CoTMPyP/Sr_2_Nb_3_O_10_ nanocomposite	voltammetric	electrode	50~3250	10.6	[37]
Carbon dots	spectrofluorometric method	fluorometric	5~50	3.2	[38]
Carbon dots	on-off-on	fluorometric	0~200	0.35	[39]
Carbon dots	on-off-on	fluorometric	5~350	3.11	[40]
Carbon dots	spectrofluorometric method	fluorometric	0~1000	0.026	This work

**Table 3:** Detected ascorbic acid (AA) concentration in animal serum.

No.	Found (μmol/L)	Added (μmol/L)	Theoretical value(μmol/L)	Measured Value (μmol/L)	Recovery (%)	RSD (%, n = 3)
1	42.28	20	62.28	63.24	101.54	2.1
2		30	72.28	72.20	99.89	0.5
3		40	82.28	82.63	100.43	1.1

## Data Availability

Not applicable.
